# Optimising the chick chorioallantoic membrane xenograft model of neuroblastoma for drug delivery

**DOI:** 10.1186/s12885-017-3978-x

**Published:** 2018-01-04

**Authors:** Rasha Swadi, Grace Mather, Barry L. Pizer, Paul D. Losty, Violaine See, Diana Moss

**Affiliations:** 1 Department of Cellular and Molecular Physiology, Institute of Translation Medicine, University of Liverpool, Crown St, Liverpool, L69 3BX UK; 2 Department of Biochemistry, University of Liverpool, Liverpool, L6 7ZB UK; 3 Department of Paediatric Oncology, Alder Hey Children’s NHS Foundation Trust, Liverpool, L12 2AP UK; 4 Academic Paediatric Surgery, Division of Child Health, University of Liverpool, Liverpool, L12 2AP UK

**Keywords:** Neuroblastoma, Chick embryo, Retinoic acid, Drug delivery, Differentiation therapy, 3Rs, Chorioallantoic membrane

## Abstract

**Background:**

Neuroblastoma is a paediatric cancer that despite multimodal therapy still has a poor outcome for many patients with high risk tumours. Retinoic acid (RA) promotes differentiation of some neuroblastoma tumours and cell lines, and is successfully used clinically, supporting the view that differentiation therapy is a promising strategy for treatment of neuroblastoma. To improve treatment of a wider range of tumour types, development and testing of novel differentiation agents is essential. New pre-clinical models are therefore required to test therapies in a rapid cost effective way in order to identify the most useful agents.

**Methods:**

As a proof of principle, differentiation upon ATRA treatment of two MYCN-amplified neuroblastoma cell lines, IMR32 and BE2C, was measured both in cell cultures and in tumours formed on the chick chorioallantoic membrane (CAM). Differentiation was assessed by 1) change in cell morphology, 2) reduction in cell proliferation using Ki67 staining and 3) changes in differentiation markers (STMN4 and ROBO2) and stem cell marker (KLF4). Results were compared to MLN8237, a classical Aurora Kinase A inhibitor. For the in vivo experiments, cells were implanted on the CAM at embryonic day 7 (E7), ATRA treatment was between E11 and E13 and tumours were analysed at E14.

**Results:**

Treatment of IMR32 and BE2C cells in vitro with 10 μM ATRA resulted in a change in cell morphology, a 65% decrease in cell proliferation, upregulation of STMN4 and ROBO2 and downregulation of KLF4. ATRA proved more effective than MLN8237 in these assays. In vivo, 100 μM ATRA repetitive treatment at E11, E12 and E13 promoted a change in expression of differentiation markers and reduced proliferation by 43% (*p* < 0.05). 40 μM ATRA treatment at E11 and E13 reduced proliferation by 37% (*p* < 0.05) and also changed cell morphology within the tumour.

**Conclusion:**

Differentiation of neuroblastoma tumours formed on the chick CAM can be analysed by changes in cell morphology, proliferation and gene expression. The well-described effects of ATRA on neuroblastoma differentiation were recapitulated within 3 days in the chick embryo model, which therefore offers a rapid, cost effective model compliant with the 3Rs to select promising drugs for further preclinical analysis.

## Background

Neuroblastoma is a paediatric cancer derived from the sympathoadrenal lineage and is thought to originate from undifferentiated neuroblasts [1]. Treatment has advanced over the last decade or more and now includes immunotherapy and differentiation therapy alongside conventional chemotherapy, radiotherapy and surgery. Overall, survival for patients with high risk neuroblastoma tumours is poor (< 50%), thus crucially indicating a need to develop additional therapies [2, 3]. Whilst many agents tested in vitro look promising, remarkably few are as successful in preclinical models or eventually patients. The most common model used for screening potential drugs is the mouse xenograft model where neuroblastoma cells are introduced either subcutaneously or orthotopically. Mouse models are expensive and time consuming hence there is a need for additional models. These models should be rapid, cost effective and NC3Rs compliant in order to contribute to the identification of novel therapies which have the potential to progress to successful preclinical/clinical trials and ultimately have a significant impact on the disease.

The chick chorioallantoic membrane (CAM) has been used for many years to support the growth of tumours including neuroblastoma [4]. It has been especially attractive as a model for studying angiogenesis due to the accessibility and visibility of the blood vessels drawn in to support tumour growth. Drugs to investigate and manipulate angiogenesis have been supplied in various formats including within plastic rings and gelatin sponges [5]. The ability of cells to form tumours on the CAM has also been used to investigate tumour biology such as the ability of tumour cells to invade and metastasise into the embryo [6–8] and most recently the CAM tumour model is increasingly finding a use as a platform to analyse the effectiveness of anticancer drugs on invasion and metastasis [9–11].

One characteristic feature of neuroblastoma is its unusually high rate of spontaneous regression and this may be connected to the susceptibility of tumour cells to differentiate. Indeed tumours with a differentiating histology and markers of mature neurons such as TrkA are low risk whilst tumours with undifferentiated histology are high risk [12, 13]. A small number of genetic mutations have been identified in neuroblastoma tumours, the first and best characterised is amplification of a variable sized amplicon containing the MYCN gene [14]. A number of neuroblastoma cell lines (typically MYCN-amplified (MNA)) have been shown in culture to slow or cease cell division and begin to extend axons in response to retinoic acid (RA). We have previously shown similar differentiation responses by the MNA cell lines Kelly and SK-N-BE2(C) triggered by the embryonic environment of the chick [15]. Thus differentiation therapy is a promising approach for treating high risk neuroblastoma and whilst some tumours and cell lines remain resistant to RA, MNA cell lines generally respond well.

Here we have used ATRA in culture as a proof of principle to validate suitable assays and timescale of response of tumours formed on the chick CAM. We show that ATRA reduces cell proliferation and increases differentiation of MNA Neuroblastoma tumours within 3 days thus establishing the CAM tumour model as a suitable in vivo model for screening new differentiation therapies.

## Methods

### Cell culture

SK-N-BE(2)C (human NB, ECACC No. 95011817) and IMR-32 (human NB, ECACC No. 86041809) were grown in DMEM (Life Technologies), 10% Foetal Bovine Serum (Biosera, East Sussex, UK), 100 U/ml penicillin,100 μg/ml streptomycin (Sigma, P0781) and 1% Non-Essential Amino Acids (Sigma, M7145). They were maintained at 37 °C with 5% CO_2_ in humidified incubator. Passaging was carried out using 0.05% Trypsin/EDTA (Sigma Aldrich) as required. Cell lines were transduced with green fluorescent protein (GFP) lentivirus as described previously [7, 15].

### Morphology analysis and cell proliferation assays

1 × 10^4^ of BE(2)C cells and IMR32 cells were plated onto coverslips in a 24 well plate, incubated for 18-24 h. Medium containing either 10 μM RA, 4 μM of MLN8237 or DMSO alone 0.06% or 0.04% final concentration was added and cells were analysed after 72 h of incubation. To assess the morphology of cells, images of cells were obtained using an inverted microscope (Leica DMIRB) prior to fixation. For immunocytochemistry, coverslips were removed from wells and fixed with 4% paraformaldehyde for 10 min, blocked with 1% BSA, 0.1% Triton X100 in 0.12 M phosphate pH 7.4 for 30 min and stained overnight at 4 °C with 1:50 dilution of Ki67 (Abcam ab16667) followed by 1:500 Goat anti rabbit Alexa 594 (Life Technologies) for one hour at room temperature both diluted in blocking buffer. Cell nuclei were stained with DAPI. Proliferating cells were quantified by Ki67 staining and normalised to the total number of nuclei stained by DAPI. At least three fields per coverslip and 3 coverslips per experiment were counted and a minimum of 300 cells per condition.

### Chick embryo CAM assays

Fertilised white leghorn chicken eggs were obtained from Lees Lane Poultry, Wirral, or Tom Barron, Preston, UK. Eggs were incubated at 38 °C and 35–40% humidity and windowed at E3 as described previously [15]. GFP-labelled cells were initially seeded onto the CAM as tumourspheres, in matrigel or as a cell suspension. A cell suspension of 2 × 10^6^ in 5 μl of DMEM seeded onto a slightly injured CAM was found to be most efficient [7]. The CAM was injured by laceration with a pipette tip or traumatisation using a strip of sterile lens tissue causing small bleed [16]. Traumatisation was found to be the most reproducible method and was used for all experiment. To further enhance the efficiency of tumour formation 5 μl of 0.05% trypsin 0.5 mM EDTA was added immediately prior to the addition of cells. For confocal analysis, 10% GFP with 90% unlabelled cells were used to facilitate observing any morphological changes inside the tumours. Eggs were resealed and incubated until E11 [17].

### Drugs administration

Embryos were treated either topically to the CAM or by injection into the allantoic cavity between E11 and E13. ATRA was used at 10 μM and 100 μM for 3 days at E11, E12 and E13 or 40 μM was used at E11 and E13. Concentration was determined based on the volume of an egg of 45 ml. 2.8 μl, 11.25 μl or 28 μl DMSO diluted to 200 μl in PBS was injected into control embryos. Embryos were dissected on E14 and tumours analysed.

### Quantitative PCR

In vitro samples: Each cell line was seeded at a density of 2 × 10^6^ per 75cm^2^ flask and after 24 h, medium was replaced with fresh medium containing either ATRA (10 μM) or MLN8237 (4 μM) or DMSO. Every 48 h the medium was replaced with fresh medium containing RA, MLN8237 or DMSO. After 3 or 6 days, RNA was extracted using RNA mini Kit (QIAGEN) according to manufacturer’s instructions. qPCR was carried out on CFX Connect (Biorad) thermocycler using iTaq Universal SYBR green mix (Biorad) 0.5 μM primers and up to 2 μl cDNA for 35 cycles. An annealing temperature of 60 °C was used for all primer pairs and three technical replicates and three biological replicates were carried out for each sample. qPCR data analysis was carried out using Bio-Rad CFX Manager 3.0 software. Normalised relative expression of target genes was calculated using the ΔΔCq analysis mode. A list of the primers used is provided in Table 1.

**Table 1:** List of primers used for qPCR analysis

Gene	Gene name	Forward 5′-3′	Reverse 5′-3′
UBC	ubiquitin C	ATTTGGGTCGCGGTTCTTG	TGCCTTGACATTCTCGATGGT
HPRT1	hypoxanthine phosphoribosyltransferase 1	TGACACTGGCAAAACAATGCA	GGTCCTTTTCACCAGCAAGCT
GAPDH	glyceraldehyde-3-phosphate dehydrogenase	AATCCCATCACCATCTTCCA	TGGACTCCACGACGTACTCA
ROBO2	roundabout, axon guidance receptor, homolog 2	GATGTGGTGAAGCAACCAGC	TGGCAGCACATCTCCACG
STMN4	stathmin-like 4	CCTAGCAGAGAAACGGGAACA	GGCGTGCTTGTCCTTCTCTT
KLF4	Kruppel-like factor 4	CGCCGCTCCATTACCAAGAGC CGGTCGCATTTTTGGCACTG	CGGTCGCATTTTTGGCACTG
MYCN	Neuroblastoma-derived v-myc avian myelocytomatosis viral related oncogene	CACAAGGCCCTCAGTACCT	ACCACGTCGATTTCTTCCTCT

In-vivo tumours: Tumours were harvested from the CAM, rinsed in phosphate-buffered saline (PBS), then transferred into RNAlater solution (QIAGEN), and stored at initially at 4 °C or −20 °C for longer term storage prior to RNA extraction. Tissue was first removed from the RNAlater and transferred to a clean RNase free falcon tube. Liquid nitrogen was used to freeze the tissue before a pestle and mortar was used to disrupt it. RNA was then extracted using RNA mini Kit (QIAGEN). qPCR was performed as described above.

### Immunohistochemistry

Tumours which were harvested for paraffin embedding were fixed overnight in 10% neutral buffered formalin and embedded in paraffin using standard protocols. Prior to staining, the slides underwent deparaffination and high temperature antigen retrieval using a DAKO PT link. Following antigen retrieval, the slides were incubated in EnvisionTM FLEX Wash Buffer (1× working solution pH 7.67; DAKO, K8007) for 5 mins prior to loading onto the DAKO Autostainer (K8012). Sections were incubated for 30 min with Ki67 antibody (1:200) (DAKO M7240) in 5% BSA in Tris Buffered Saline followed by goat anti-mouse HRP (Abcam) and staining with 3,3′-diaminobenzidine. Haematoxylin staining was performed on all the slides and some slides were also stained with eosin to assist in distinguishing between tumour and chick nuclei. A total of 12 fields from 3 slides were counted per tumour and at least two tumours per condition were analysed.

### Morphology analysis

Tumours required for confocal imaging were fixed in 4% paraformaldehyde for one hour, trimmed into small pieces <2mm^3^ and mounted into slides using DAKO mounting medium. The images were observed using the Leica DMIRE2 microscope at X40 objective to assess the morphology of cells within the tumours.

### Statistical analysis

Statistical significance was computed using Student’s t-test or one-way ANOVA followed by a post-hoc tukey test using SPSS. All data are presented as mean + S.E.M. (standard error of the mean).

## Results

### Assessment of ATRA effects by measuring cell proliferation and expression of differentiation markers

**Fig. 1:**
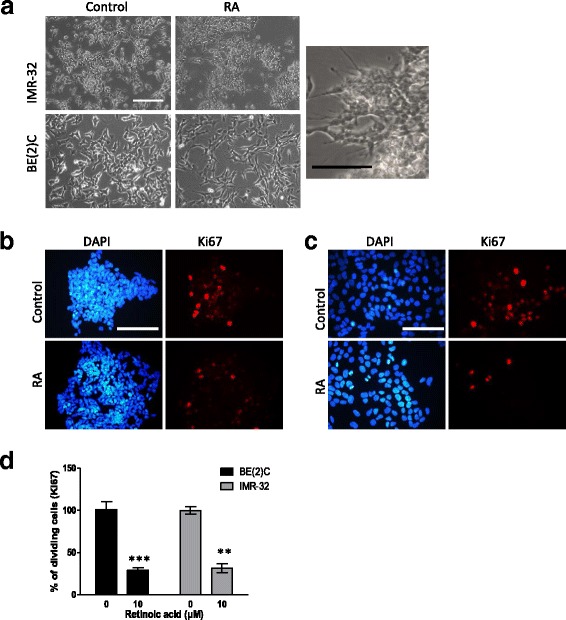
ATRA promotes a reduction in cell proliferation and change in morphology in IMR-32 and BE(2)C cells. **a** Morphological changes in IMR-32 and BE(2)C after 3 days of treatment with 10 μM ATRA (RA), an enlarged view of IMR-32 cells is displayed showing a number of cellular extensions of variable length. **b** DAPI stained (blue) and Ki67 stained (red) BE(2)C cells following three days of treatment with 10 μM ATRA or DMSO (control). **c** DAPI stained (blue) and Ki67 stained (red) IMR-32 cells following three days of treatment with 10 μM ATRA or DMSO. **d** Graph to show the reduction in cell proliferation following treatment with ATRA. Each bar represents three biological replicates and at least 9 fields per experiment. ** *p* < 0.01 and ***p < 0.001. Error bars are standard error (SE). Scale bar is 100 μM

**Fig. 2:**
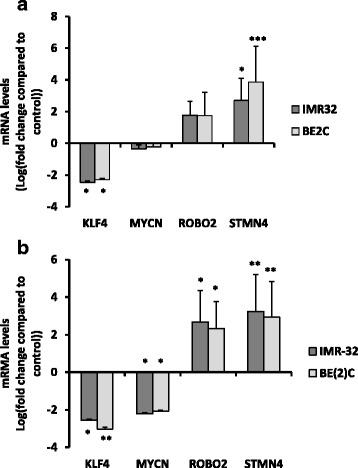
ATRA up-regulates differentiation markers ROBO2 and STMN4 and down-regulated the stem cell marker KLF4 in IMR-32 and BE(2)C cells. Relative mRNA levels for the target genes was determined by qPCR. **a** Graph displays the level of target gene expression in BE(2)C and IMR-32 cells after 3 days of ATRA (RA) treatment (10 μM) relative to DMSO treated cells, **b** shows the change in expression after 6 days of treatment with RA. Each bar in the graph represents the mean of technical replicates and three biological repeats. Error bars are SE (T-test). * *p* < 0.05, ** *p* < 0.01, *** *p* < 0.001

The effect of ATRA on MNA neuroblastoma cells has been well characterised in terms of morphology and immunofluorescence of differentiation markers [18–20]. We wished to develop assays that would enable us to quantify the extent of differentiation upon drug treatment and be suitable to compare effects in culture and in CAM tumours. Differentiation usually goes in parallel with a decrease in proliferation, which can be measured by Ki67 staining. The expression of differentiation and stem cell markers allows direct quantification of the differentiation process and can be assessed by qPCR. Two MNA cell lines BE2C and IMR32 were selected as they respond well to RA. In cell culture, 10 μM ATRA treatment for 3 days prompted the expected change in morphology (Fig. 1a) and a 65% decrease in the proliferation rate in both cell lines (Fig. 1b–d). The expression of two differentiation markers (STMN4 and ROBO2) and one stem cell marker (KLF4) was further analysed by qPCR. These three markers have previously been shown to exhibit significant changes in IMR32 cells in response to ATRA by qPCR [21]. Here, STMN4 increased more than 6 fold (*p* < 0.001) and KLF4 decreased by 5.5 fold (*p* < 0.05) in IMR32 treated with ATRA for 3 days (Fig. 2a). A non-significant increase in ROBO2 and small decrease in MYCN expression was also observed. The results with BE2C cells were similar (Fig. 2a). To test whether the changes with ROBO2 and MYCN would become significant we treated both cells lines with ATRA for 6 days in culture. By this stage ROBO2 was significantly up regulated in both cell lines and MYCN was significantly down regulated confirming the trends observed at three days (Fig. 2b).

### Comparison of ATRA effects with an aurora a kinase inhibitor, MLN8237 on neuroblastoma cell differentiation

**Fig. 3:**
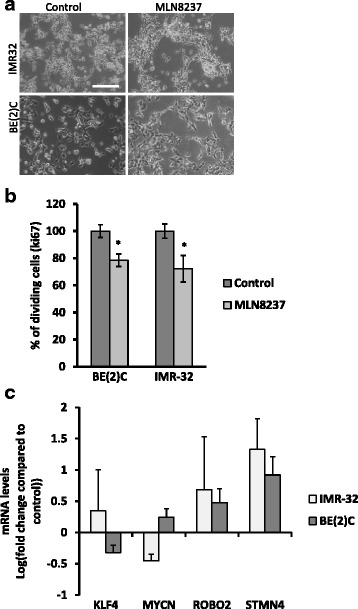
The effect of MLN8237 treatment on IMR-32 and BE(2)C cells. **a** Morphological changes of both cell lines after 3 days of 4 μM MLN8237 treatment. **b** shows the percentage change in proliferating cells in IMR-32 and BE(2)C following treatment with 4 μM MLN8237 for 3 days. **c** qPCR analysis of changes in target gene expression following 3 days of treatment. Each bar represents the mean of 3 technical replicates and 3 biological replicates. Although the trend in the changes of gene expression are the same as for ATRA the fold change is smaller and does not reach significance. Error bars (SE). Scale bar 100 μM

Aurora A kinase has been shown to stabilise MYCN [22] and early results in preclinical models have proven promising with data suggesting that MLN8237 might reduce MYCN protein levels, decrease cell proliferation and increase differentiation of Neuroblastoma cells [23, 24]. We tested whether this drug was more effective than RA, prior to further validation of drug delivery to the CAM tumours. We tested 1, 4 and 10 μM MLN8237 in both cell lines. Whilst 10 μM showed some toxicity, 4 μM showed some change in morphology after 3 days (Fig. 3a). MLN8237 also reduced cell proliferation by 22% (BE2C) and 24% (IMR32) a much smaller extent than with ATRA (Fig. 3b). The qPCR results showed no significant change for any of the markers after 3 days treatment in culture although similar trends as for ATRA were observed (Fig. 3c). ATRA was therefore used for subsequent in vivo experiments.

### Optimisation of tumour formation onto the CAM, for a wide range of neuroblastoma cell lines

**Fig. 4:**
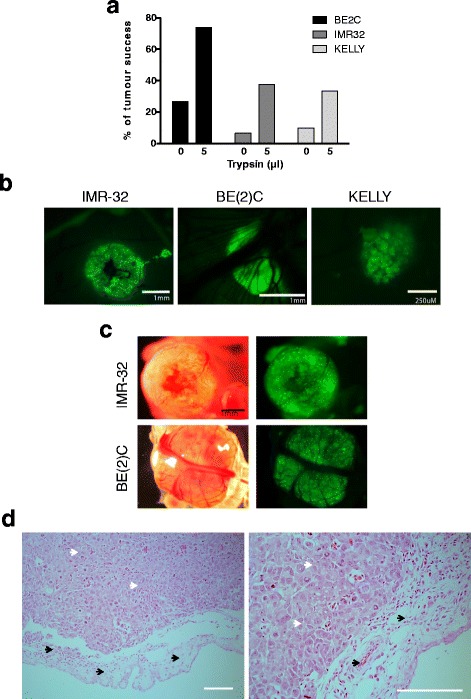
Tumour formation in chick embryo model. **a** Tumour formation in the chick embryo with and without the addition of trypsin/EDTA. Percentage tumour formation was calculated by dividing the number of eggs with tumours at E14 by the total number of embryos surviving until that time (between 30 and 40 embryos for each cell line and condition were analysed). **b** GFP images showing IMR-32 BE(2)C and Kelly tumours that have formed under the CAM. Note the smaller size of the Kelly tumours. **c** dissected IMR-32 and BE(2)C tumours viewed with GFP fluorescence with their corresponding bright field image. Note the chick CAM tissue that surrounds each tumour. **d** H&E staining of BE(2)C tumour FFPE sections. Black arrows indicate chick tissue. White arrows indicate tumour tissue. Scale bar is 250 μM

Some cell lines efficiently form tumours on the CAM whilst others, including BE2C and IMR32, do so less frequently [25]. This was surprising since MNA cells are thought to be both aggressive and invasive cells and BE2C and Kelly cells readily extravasate into the embryo following intravenous injection [15]. SKNAS cells form tumours efficiently [7] and BE2C cells mixed with SKNAS cells also formed tumours. However BE2C or IMR32 cells alone more typically formed a sheet of dried cells on the surface of the CAM (data not shown), suggesting that the cells lacked invasive potential. This prompted us to test treating the CAM surface with trypsin immediately prior to adding the cells to facilitate invasion and this indeed improved the efficiency of tumour formation for BE2C cells to more than 70% (Fig. 4a). Trypsin treatment also improved the efficiency of tumour formation for IMR32 and Kelly cells although these remained less efficient than BE2C cells. Tumours formed beneath the surface of the CAM became visible under the fluorescent stereomicroscope within 3–4 days and reached 1-5 mm in size by E14 (Fig. 4b-c). Tumours had clearly recruited blood vessels from the surrounding CAM and were highly vascularised although the IMR32 tumours were less vascularised than the BE2C. The histology of the tumours formed reflected the histology of patient tumours suggesting the tumours are a good model for preclinical analysis (Fig. 4d).

### RA promotes differentiation and reduces proliferation of BE2C and IMR32 tumours

**Fig. 5:**
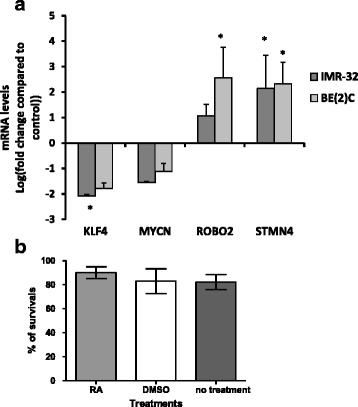
ATRA promotes the differentiation of tumours without affecting chick embryo survival. **a**. qPCR analysis of changes in relative target gene expression following daily treatment of 100 μM ATRA at E11, 12 and 13. Each bar in the graph represents the mean of three biological repeats. ROBO2 and STMN4 show statistical significant changes for BE(2)C and KLF4 and STMN4 show significant changes for IMR32. *p < 0.05. Error bars are SE. **b**. Neither DMSO (28 μl injections X3) nor ATRA (100 μM injections X3) affected the survival of the chick embryos (*n* > 30 for all conditions)

Tumours could be reliably observed by E11 so ATRA treatment was initiated at E11 and repeated at E12 and E13. The volume of eggs was approximately 45 ml and ATRA was added to give a final concentration of 100 μM which was equivalent to 30 mg/kg used for treating mouse xenograft tumours [26]. ATRA is insoluble in aqueous solutions however survival of embryos is compromised by more than 100 μl of DMSO and by the addition of 100% DMSO [27]. Hence the ATRA diluted in maximally 28 μl of DMSO was further diluted up to 200 μl with PBS to form a colloidal suspension. Topical addition of the suspension was used successfully in some experiments however in some cases not all the ATRA re-dissolved. Hence, in later experiments the colloidal suspension was injected into the allantoic sac where it reproducibly dissolved within a few hours, aided by the movements of the chick embryo. Tumours treated with and without ATRA were analysed for changes in the differentiation markers and also for MYCN (Fig. 5a). For IMR32 cells, the results were similar to those observed in culture, with KLF4 down regulated 4.2 fold and STMN4 upregulated 4.4 fold (*p* < 0.05). ROBO2 and MYCN showed an appropriate trend that although it was not statistically significant. For BE2C cells both ROBO2 and STMN4 were significantly upregulated (5.9 fold and 5.0 fold respectively; *p* < 0.05) and KLF4 and MYCN were down regulated by 3.4 and 2.2 fold respectively although these latter results were not statistically significant. Survival of the embryos was unaffected by the introduction of ATRA compared to either 28 μl of DMSO per injection or no treatment (Fig. 5b).

**Fig. 6:**
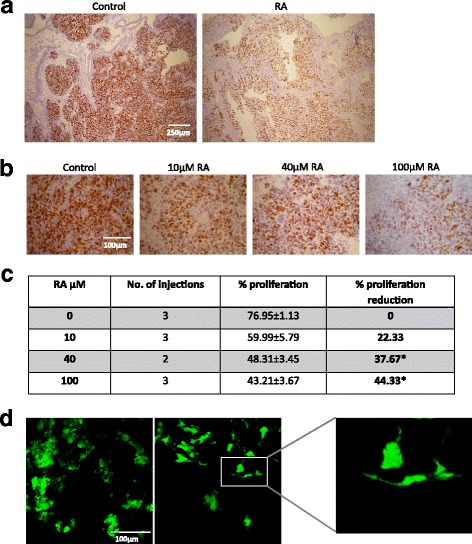
Retinoic acid reduces cell proliferation and alters cell morphology in tumours. **a**. FFPE sections stained with Ki-67. Tumours were treated with 100 μM ATRA at E11, E12 and E13 and compared to the control which was treated with the equivalent volume of DMSO, **b**. FFPE sections stained with Ki-67. Treatments were 10 μM ATRA (10 μM at E11, E12 and E13), 40 μM ATRA (40 μM at E11 and E13) and 100 μM ATRA (100 μM at E11, E12 and E13). Note the decreasing number and staining intensity of the cell nuclei as the concentration of ATRA is increased. **c**. Table showing the quantification of the proliferative cells in BE(2)C tumours after different ATRA treatments. Results suggested that both 40 μM of ATRA (2 injections) and 100 μM (3 injections) reduces the number of proliferative cells significantly (*p < 0.05) compared to the control. **d** confocal image of tumour treated with 40 μM (×2) ATRA or DMSO. Tumours were formed from BE(2)C cells of which 10% expressed GFP. Morphological changes were observed in some of the ATRA treated tumour cells

The effect of ATRA on cell proliferation of BE2C cells within the tumour was also assayed. The percentage of dividing cells was reduced by 43% compared to the DMSO control (Fig. 6a). Treatment of tumours with 100 μM every 24 h was a considerably higher dose than the 10 μM every 48-72 h used in culture. We therefore tested the effect of lower doses of ATRA. As shown in Fig. 6b, 10 μM ATRA (3 doses) reduced proliferation by 22% (not significant) while 40 μM (2 doses at E11 and E13) reduced proliferation by 37% (*p* < 0.05) (Fig. 6b and c). Thus four times the dose used in culture was sufficient to produce statistically significant results in vivo (Fig. 6c).

Finally we sought to test whether 40 μM ATRA would also change the morphology of cells within the tumour as is typically observed in culture. For this experiment, only 10% of the cells within the tumour expressed GFP with the remaining 90% were unlabelled BE2C cells. Confocal images of tumours treated with ATRA or DMSO are shown in Fig. 6d. ATRA treated cells exhibited a more elongated shape with cells often having small extensions resembling neurites.

Taken together these results demonstrate, using ATRA as a proof of principle, that the chick CAM model can be used successfully for drug treatment thereby providing a platform of choice for further evaluation of drug efficiency in neuroblastoma.

## Discussion

The chick embryo has been used extensively to study development however its use for investigating cancer biology, especially its value for testing the efficacy of drugs, has been more limited to date [9–11]. The chick embryo also complies with widely accepted guidelines designed to reduce animal numbers, refine and replace animal models (the 3Rs) [28]. In our experiments we introduce cells onto the CAM at E7, the earliest time point at which the CAM is sufficiently developed, and complete experiments at E14. Hence these experiments, although in vivo*,* are not considered animal experiments under UK legislation and thus replace the use of animals.

Many cell lines form tumours on the CAM however some do not [25, 29] a feature we have also observed with Neuroblastoma cell lines. Tumour cells need to invade through the epithelial sheet of the CAM and this may require functioning MMPs to be secreted by the tumour cells. Whilst many Neuroblastoma secrete MMPs only SKNAS cells, of those tested, also expressed the biological activator [30]. This provides an explanation for the greater efficiency of tumour formation by SKNAS cells [7] and the rationale for the use of trypsin to enhance tumour formation for IMR32, Kelly and especially BE2C cells. Use of trypsin may enhance the use of the CAM tumour model by expanding the range of cell lines that will form tumours efficiently.

Drugs can be introduced to the embryo and extra embryonic tumours by topical addition, intravenous (IV) injection or injection into the allantois [31]. We compared topical addition against IV injection using 5-ethynyl-2′-deoxyuridine (EdU) and found similar numbers of EdU labelled cells in the CAM tumour and the liver of the embryo within 24 h [32]. Indeed given that drugs, by design, pass into and out of blood vessels it would be surprising if there was a significant difference between the two delivery methods. Since IV injections are technically more difficult we did not pursue this as a delivery method. For water-insoluble drugs such as ATRA we found that the allantoic sac provided the optimum method of delivering drugs since colloids have the opportunity to redissolve and be distributed through the egg aided by the movements of the embryo. One limitation in introducing drugs into embryos is their solubility. Water soluble drugs are not a problem however DMSO is a typical solvent for water-insoluble drugs and chick embryos will tolerate no more than 100 μl of DMSO [27] and do not tolerate the introduction of 100% DMSO. We circumvented the insolubility of ATRA by forming a DMSO:PBS ATRA colloidal mixture and injecting this into the allantoic sac.

RA was used for our experiments since MLN8237 was less effective as a differentiation agent for culture BE2C and IMR32 cells despite reports of good results with tumours formed by the TH-MYCN mouse [23] and xenografted mice [24]. Tumour formation for BE2C cells can be reproducibly observed by fluorescent microscopy by E11 so ATRA injections commenced from E11. ATRA is used in culture at 10 μM replenished every 48-72 h whilst in mice a daily dose of approximately 100 μM (30 mg/kg) is delivered by oral gavage [26]. Initial experiments were carried out using this higher dose about 10 fold greater than used in vitro*.* Embryos tolerated this dose well and changes in differentiation markers were similar to cultured cells while the reduction in proliferation was somewhat less than observed in vitro. Nevertheless we were interested to determine the dose required to observe statistically significant effects of ATRA and whilst a daily dose of 10 μM ATRA showed the appropriate trend it required two doses of 40 μM ATRA at E11 and E13 to give statistically significant changes in proliferation and a change in cell morphology. This fourfold increase over the concentration used in culture may be due to sequestration of the ATRA by the receptors present in cells in the embryo [33] thus potentially reducing the effective concentration. In addition, the cells within the tumour maybe less responsive than those in culture; perhaps reflecting the differing microenvironment [34].

RA is already established as an effective drug for clinical use [35] however some tumours and cell lines are resistant and for others the response is incomplete. Here we have established a method of enhancing tumour development on the CAM, delivering water-insoluble drugs to the tumours and three outcomes that confirm differentiation of cells (qPCR of differentiation markers, reduction in proliferation and change in cell morphology). Chick embryos develop rapidly with a window of only 7 days between a sufficiently developed CAM (E7) and the age embryos come under UK Home Office regulation (E14). Nevertheless tumours can form on the CAM and respond to drug treatments in this time window making the model highly time efficient. It is especially useful for analysing the cellular response to drug treatment as changes in gene expression, leading to different cell behaviours typically occur on a time scale of hours to days. These changes rather than, for example, changes in tumour size suit the short term nature of the model. We can now extend our results in order to rapidly and cost effectively test other putative differentiation agents alone or in combination with RA. Furthermore we have recently shown that neuroblastoma cells will metastasise into the embryo following preconditioning in hypoxia [7]. It will be interesting to discover whether ATRA or other differentiation agents can reverse the effect of hypoxia and reduce or inhibit the metastasis of Neuroblastoma cells.

## Conclusions

40 μM ATRA (4 times the concentration used in culture), injected into the allantoic sac of a chick embryo, reduces proliferation of neuroblastoma cells in tumours formed on the chick CAM within three days and changes cell morphology. 100 μM ATRA promotes changes in differentiation markers within three days. These results confirm that ATRA treatment of tumours formed on the chick CAM are comparable to those observed in mouse xenograft tumours [36]. Thus we have established an efficient and robust protocol for using tumours formed on the chick embryo CAM to test novel therapies. The model is highly cost effect compared to the mouse xenograft model, is rapid and 3Rs compliant.

## Abbreviations

ATRA: All-trans retinoic acid
BPS: Phosphate buffered saline
BSA: Bovine serum albumin
CAM: Chorioallantoic membrane
cDNA: Complementary deoxyribonucleic acid
DAPI: 4′,6-diamidino-2-phenylindole
DMEM: Dulbecco’s modified eagle medium
DMSO: Dimethyl sulfoxide
EdU: 5-ethynyl-2′-deoxyuridine
FFPE: Formalin-fixed paraffin-embedded
GAPDH: Glyceraldehyde-3-phosphate dehydrogenase
GFP: Green fluorescent protein
HPRT1: Hypoxanthine phosphoribosyltransferase 1
HRP: Horseradish peroxidase
KLF4: Kruppel-like factor 4
MMPs: Matrix metalloproteinases
MNA: MYCN-amplified
MRNA: Messanger RNA
MYCN: Neuroblastoma-derived v-myc avian myelocytomatosis viral related oncogene
NB: Neuroblastoma
QPCR: Quantitative PCR
RA: Retinoic acid
ROBO2: Roundabout, axon guidance receptor, homolog 2
STMN4: Stathmin-like 4
TrkA: Tropomyosin receptor kinase A
UBC: Ubiquitin C
